# Sampling with poling-based flux balance analysis: optimal versus sub-optimal flux space analysis of *Actinobacillus succinogenes*

**DOI:** 10.1186/s12859-015-0476-5

**Published:** 2015-02-18

**Authors:** Michael Binns, Pedro de Atauri, Anestis Vlysidis, Marta Cascante, Constantinos Theodoropoulos

**Affiliations:** 1 School of Chemical Engineering and Analytical Science, University of Manchester, Manchester, M13 9PL UK; 2 Department of Biochemistry and Molecular Biology, Faculty of Biology and Institute of Biomedicine (IBUB), University of Barcelona, 08028 Barcelona, Spain; 3 Currently at: Department of Chemical Engineering, Hanyang University, 222 Wangsimni-ro, Seongdong-gu, Seoul 133-791 Republic of Korea

**Keywords:** Flux sampling, Optimisation, Flux balance analysis

## Abstract

**Background:**

Flux balance analysis is traditionally implemented to identify the maximum theoretical flux for some specified reaction and a single distribution of flux values for all the reactions present which achieve this maximum value. However it is well known that the uncertainty in reaction networks due to branches, cycles and experimental errors results in a large number of combinations of internal reaction fluxes which can achieve the same optimal flux value.

**Results:**

In this work, we have modified the applied linear objective of flux balance analysis to include a poling penalty function, which pushes each new set of reaction fluxes away from previous solutions generated. Repeated poling-based flux balance analysis generates a sample of different solutions (a characteristic set), which represents all the possible functionality of the reaction network. Compared to existing sampling methods, for the purpose of generating a relatively “small” characteristic set, our new method is shown to obtain a higher coverage than competing methods under most conditions.

The influence of the linear objective function on the sampling (the linear bias) constrains optimisation results to a subspace of optimal solutions all producing the same maximal fluxes. Visualisation of reaction fluxes plotted against each other in 2 dimensions with and without the linear bias indicates the existence of correlations between fluxes. This method of sampling is applied to the organism *Actinobacillus succinogenes* for the production of succinic acid from glycerol.

**Conclusions:**

A new method of sampling for the generation of different flux distributions (sets of individual fluxes satisfying constraints on the steady-state mass balances of intermediates) has been developed using a relatively simple modification of flux balance analysis to include a poling penalty function inside the resulting optimisation objective function. This new methodology can achieve a high coverage of the possible flux space and can be used with and without linear bias to show optimal versus sub-optimal solution spaces. Basic analysis of the *Actinobacillus succinogenes* system using sampling shows that in order to achieve the maximal succinic acid production CO_2_ must be taken into the system. Solutions involving release of CO_2_ all give sub-optimal succinic acid production.

**Electronic supplementary material:**

The online version of this article (doi:10.1186/s12859-015-0476-5) contains supplementary material, which is available to authorized users.

## Background

The identification of a reaction network and the determination of reaction fluxes and metabolic concentrations at typical steady-state conditions are key first steps towards understanding the metabolic processes in an organism. Reaction networks can be constructed through extracting information from the genome and inserting corresponding reactions found in databases [1]. For example this should include the identification of: enzymes known to be present in a given organism, reactions associated with those enzymes and metabolites involved or required by those reactions. Subsequently, this list of reactions should be verified and reactions should be added or removed based on knowledge gathered from the literature (a complicated procedure). This process can be supplemented using reaction prediction tools [2-4] to fill in any gaps in the network where unknown or unfamiliar reactions are occurring. Using experimental measurements where available for the significant reaction fluxes going in and out of the system (external fluxes) flux balance analysis can be applied to compute the remaining internal and external fluxes using linear programming with steady-state constraints. Flux balance analysis, however, is limited in that optimisation with a certain linear objective function will give a single optimal solution. Nevertheless, it is well known that this leaves a large degree of uncertainty in the internal reaction fluxes [5,6] when there are reaction cycles and branching reactions present. In particular, for genome-scale models Mahadevan and Schilling [6] and Soh et al. [7] suggest that the existence of alternative solutions for flux balance analysis (FBA) resulting from this uncertainty is a key challenge and Soh et al. suggest this could be resolved with the identification of characteristic flux distributions explaining the observed steady-state behaviour of the phenotype. In addition, it is possible to remove this uncertainty by providing additional constraints [8]. Yet, even with extra constraints FBA still aims to compute a single flux distribution; in most cases there are a large number of solutions, which are missed through the additional assumptions made in order to force the system to a single solution.

Flux variability analysis can be used to quantify the size of the uncertainty and the range of possible flux values that these internal reactions can achieve while still giving the same maximum or minimum flux values obtained through flux balance analysis [6,9]. Although the true flux values for internal reactions remain unknown, a number of attempts have been made to provide different possible sets of values. These include the calculation of pathways with elementary flux modes [10,11] and extreme pathways [12] or with the use of mixed integer linear programming (MILP) [13,14]. For cases with large reaction networks including a large degree of uncertainty it is infeasible to compute all possible solutions using these combinatorial algorithms as the number of solutions increases exponentially with the size of the network. However, by considering only the optimal flux sub-space and through the identification and removal of redundancy (particularly fixed fluxes) Kelk et al. [15] have shown that it is possible to enumerate all possible solutions (within the optimal sub-space) for large reaction systems as vertices, rays and linealities which describe the pathways and loops in the network. While these combinatorial and pathway generating methods provide an interesting and informative selection of flux distributions they are also limited in that they do not consider the entire flux space, but typically focus on its extreme limits. To consider the entire region of feasible solutions, sampling methods can be used to produce a set of flux distributions covering the entire convex flux space [16-18]. Existing methods for sampling the convex flux space include the Monte Carlo algorithm called “hit-and-run” [16,19] and the improved “artificial centering hit-and-run” (ACHR) algorithm [17]. As noted by Schellenberger and Palsson [5] the ACHR algorithm is the basis for most publications involving flux sampling. These hit-and-run algorithms aim to give a uniform distribution, or a distribution that matches a specified probability distribution. Their main limitation is that they do not guarantee a high coverage and may require a very large number of steps to achieve a desired uniform coverage in a high dimensional system [5].

Sampling has been applied for a number of aims including calculation of available flux space volume [18,20] and also for the identification of exact or approximate correlations between reaction fluxes [20]. The existence of alternate solutions has also been shown to have an effect on gene-knockout studies, which are used to test if an organism contains the necessary redundancy to overcome the loss of a gene [6]. In addition there are several works where a uniform sample of flux distributions have been used to perform some statistical analysis giving histograms (probability distributions) for individual reaction fluxes [18,20,21]. However these works might use a very large sample (e.g. 250000–1000000 flux distributions) to guarantee a uniform coverage and their results are based on the assumption that all flux distributions have equal probability.

The aim of this work is to develop a methodology for sampling which gives a “small” characteristic set of flux distributions representing the full range of capabilities of the network. This new methodology uses optimisation to maximise the difference between flux distributions within the sample in order to find a diverse sample of “adequately different” solutions, which yield the maximum coverage with the minimum number of flux distributions. Compared with existing methods which might converge slowly to a uniform coverage this new method converges quickly even for high dimensional systems. Hence, compared to our method a hit-and-run algorithm would require a significantly larger number of steps (or multiple steps per flux distribution) to achieve a similar level of detail.

Our methodology generates results exploiting a *linear bias*, which constrains each computed solution to an optimal subspace of solutions all giving the same maximal reaction fluxes. This linear bias is implicitly included in the linear objective function. Without it the full range of possible solutions can be sampled including all the sub-optimal solutions. Comparisons of the optimal and sub-optimal solution space can give some insights into the fundamental properties of the system.

## Methods

Flux balance analysis allows the identification of theoretical limits for different reaction fluxes in a metabolic system. This is achieved through fixing measured experimental fluxes and optimising certain reaction fluxes while assuming the intermediate metabolites are held at steady state. This is typically formulated as a linear problem:

1 $$ S\cdot v=0 $$

where *S* is the stoichiometry matrix containing the mass balances for all the internal metabolites, *v* is a vector containing the reaction fluxes and equation 1 is satisfied if all the internal metabolites are maintained at steady state.

### Poling sampling

Our new methodology involves modifying the formulation of flux balance analysis in order to generate multiple different solutions. This is accomplished through the addition of a penalty function to the objective function while the constraints (Eq. 1) used for flux balance analysis remain the same. The penalty function used here is the same as that utilised in the poling method [22], which is commonly employed in order to generate different 3D conformations in molecules. Here we have applied it in order to generate different flux distributions. The basic form of the penalty function is the one implemented by Smellie et al. [22].

2 $$ {\mathrm{F}}_{\mathrm{pole}}={\mathrm{W}}_{\mathrm{pole}}{\displaystyle {\sum}_{\mathrm{i}=1}^{\mathrm{n}}\frac{1}{{\left({\mathrm{D}}_{\mathrm{i}}\right)}^{\mathrm{N}}}} $$

3 $$ {\mathrm{D}}_{\mathrm{i}}={\left(\frac{\left({\displaystyle {\sum}_{\mathrm{j}=1}^{{\mathrm{N}}_{\mathrm{v}}}{\left({\mathrm{v}}_{\mathrm{j}}\hbox{-} {\mathrm{v}}_{\mathrm{i}\mathrm{j}}\right)}^2}\right)}{{\mathrm{N}}_{\mathrm{v}}}\right)}^{1/2} $$

Hence, the total objective function is given simply by

4 $$ {\mathrm{F}}_{\mathrm{total}}={\mathrm{F}}_{\mathrm{linear}}+{\mathrm{F}}_{\mathrm{pole}} $$

Here F_linear_ is some linear objective, v_j_ is the unknown flux through the j^th^ reaction step and v_ij_ is the previously calculated flux through the j^th^ reaction in the i^th^ flux distribution. It is worth noting here that F_linear_ is the linear objective term inherited from FBA and if F_pole_ was set to zero the optimisation would be exactly the same as that of FBA. In the penalty function W_pole_ and N are poling parameters controlling the size of the penalty function with respect to the linear objective. Also, n is the current number of previously calculated flux distributions in the sample and N_v_ is the number of reaction flux steps (i.e. including only the non-zero steps for which $\left({v}_j^{max}-{v}_j^{min}\right)>1\times {10}^{-12}$ mmol g-DCW^−1^ h^−1^) in the reaction network. For the first flux distribution generated n=0 and the penalty function is equal to zero. Each subsequent flux distribution will be different due to the influence of the penalty function, which pushes the optimisation away from the n previous flux distributions. In practice we have implemented this procedure using deterministic local optimisation methods which are convenient for obtaining answers in a short time but which may miss the best possible global optimal points. If a global optimisation method was implemented then better solutions could be obtained giving a higher quality sample at the expense of a greatly increased computational time.

In order to remove the bias introduced by the linear objective we have also considered an additional objective function (Eq. 5), where F_total_ = F_linear_ for computing the first solution (*n*=0) and F_total_ = F_pole_ for all subsequent solutions. So with the exception of the first solution the sample and all possible fluxes will be computed without any bias.

5 $$ {\mathrm{F}}_{\mathrm{total}}={\delta}_{n+1,1}{\mathrm{F}}_{\mathrm{linear}}+{\mathrm{F}}_{\mathrm{pole}} $$

*δ*_*n* + 1,1_ = 1 if *n* = 0 and *δ*_*n* + 1,1_ = 0 if *n* > 0

### Constraints and flux variability

Initially all the internal reaction fluxes are considered to be unknown except for those that are determined through experiments. For practical reasons these unknown fluxes are normally given large magnitude positive upper limits and large magnitude negative lower limits. However, in cases where the reactions are split into forwards and backwards reactions one of the limits is set to zero, accordingly. Starting from these large initial limits flux variability analysis [6,9] is applied to tighten these bounds through maximisation and minimisation of each flux while maintaining steady state for the internal fluxes i.e. satisfying equation 1.

### Coverage analysis

The main aim of sampling is to produce a characteristic set of fluxes, which represent all the capabilities of the system. To maximise the effectiveness of the set of computed solutions it is desirable to cover the search space of possible fluxes in order to account for all possibilities. Hence it is also necessary to measure how well a given sample covers the available search space. An outer perimeter to the available search space is defined through flux variability analysis, by maximising and minimising each flux.

Existing criteria which can be applied to assess the quality of a generated sample such as the method of Gelman and Rubin [23] quantify the “convergence” of the sample to a specified target probability distribution (typically a uniform distribution). This is useful if a uniform distribution is required to perform additional analysis or statistics but a “converged” sample may still contain gaps (possibly with missed functionality) and to meet the criteria for convergence may require the generation of a very large sample.

However, the new sampling method described here has a different objective and the samples it generates will not necessarily give a uniform final distribution. The aim of sampling in this study is the generation of a small sample which efficiently (without redundant or very similar solutions) represents the full range of capabilities of the system (without gaps containing missed functionality). Hence, we have created a new criteria for measuring the quality of a sample which we call “coverage”.

One way to measure the coverage is to determine the maximum gap between values in a sample for each reaction flux range. This is defined as

6 $$ \mathrm{gap}\left(\mathrm{j}\right)= \max \left({\delta}_{q+1,z}\left({\nu}_j^z-{\nu}_j^q\right)\right) $$

*δ*_*q* + 1,*z*_ = 1 if *q*+1= *z, δ*_*q* + 1,*z*_ = 0 if *q*+1≠ *z*

7 $$ Coverage=1-\frac{1}{N_{nz}}{\displaystyle {\sum}_{non\mathit{\hbox{-}} zero\kern0.2em  fluxes}\frac{gap(j)}{v_j^{max}-{v}_j^{min}}} $$

**Figure 1:**
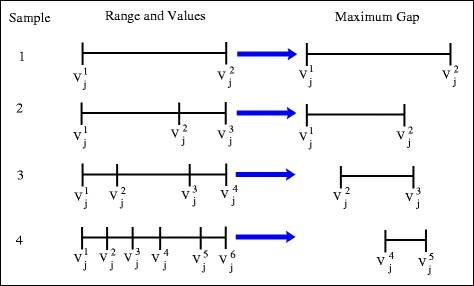
**Simple examples illustrating the maximal gap identified for the j** ^**th**^ **reaction in 4 different samples.**

Where the different reaction fluxes in a sample are first sorted from smallest to largest with the first value equal to the lower bound ${v}_j^{min}$ (the minimum value from flux variability analysis) and the final n^th^ value equal to the upper bound ${v}_j^{max}$ (the maximum value from flux variability analysis). Equation 6 identifies the largest gap (superscripts z and q refer to different flux distributions within the sample with δ_*q* + 1,z_ ensuring that gaps are found between adjacent flux values in the sequence) within the sample for each flux range for each reaction, j, and equation 7 summarises the overall coverage of the sample. Equation 7 only considers non-zero fluxes (N_nz_ is the number of non-zero fluxes) for which $\left({v}_j^{max}-{v}_j^{min}\right)> tolerance$ (in our case tolerance was set at 1 × 10^−12^) to avoid fluxes which are set to zero due to certain reactions being irreversible in the system. The meaning of the largest gap as described by equation 6 for each reaction is emphasised in Figure 1 which shows how the largest gap is extracted from a set of sample data points. It should also be clear that based on equation 7 a smaller set of (maximum) gaps will give a higher coverage of the search area.

## Results and discussion

### Case study: central metabolism of *Actinobacillus succinogenes*

**Figure 2:**
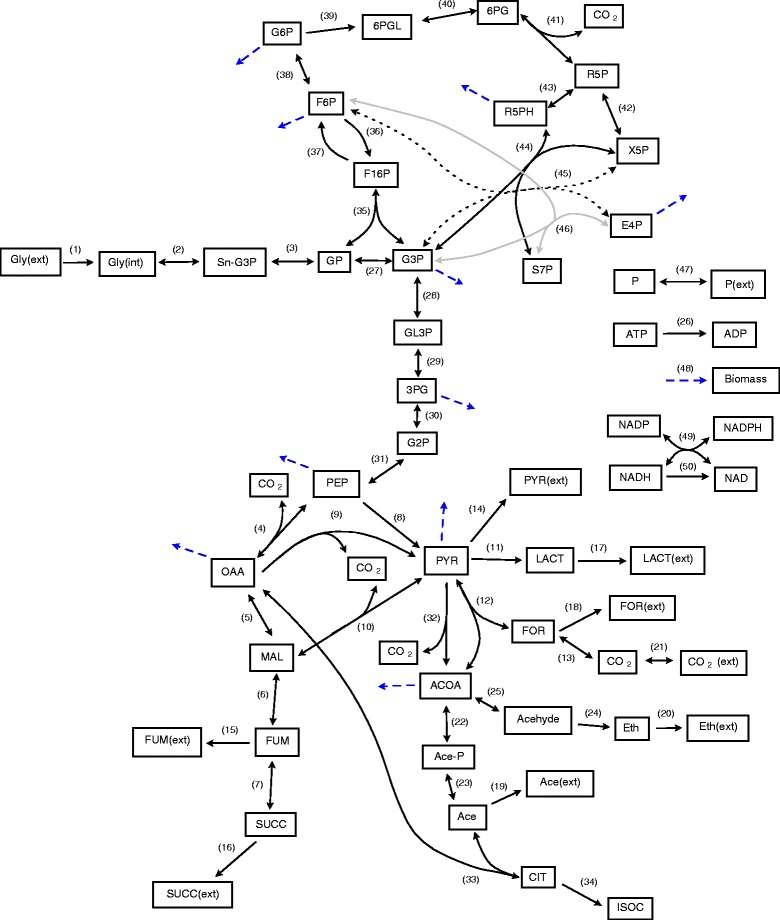
**Metabolic reaction network for** ***Actinobacillus succinogenes*** **.** Unidirectional arrows indicate steps which are considered to be irreversible, all other steps are assumed to be reversible. Short dashed arrows correspond to reaction 48 indicating the metabolic species contributing to biomass production. For simplicity, explicit consumption or production of ATP, ADP, NAD, NADH, NADPH and NADP is not included in all reactions. However these exchanges were included, e.g. ATP is consumed or produced in reactions 2, 4, 8, 23, 26, 29, 36 and 48.

*Actinobacillus succinogenes* is an organism which is known for producing succinic acid either from glucose [24-29] or glycerol [30]. Here we consider the production of succinic acid from glycerol via the metabolic reaction network shown in Figure 2. This network is constructed using information gathered from literature concerning *Actinobacillus succinogenes* [24,27,28,30-32] in addition to thermodynamics considerations [33] in order to determine the reversibility of some steps (see Table 1). The full names of metabolites and the enzymes associated with the numbered reactions are given in Additional file 1.

**Table 1:** **Reversibilities and change in Gibbs free energy of selected reaction fluxes (see Figure** 2 **)**

**Reaction number**	**∆G (KJ mol** ^**−1**^ **)**	**Reversible**
4	+1.21	YES
5	−26.04	YES
8	−19.3	NO
9	−20.52	NO
10	−5.53	YES
12	−21.13	YES
13	−12.94	YES
32	−28.76	NO
39	−22.96	NO

### Constraints and measured fluxes

A number of external fluxes were measured based on experiments carried out in batch reactors [30] giving values for glycerol uptake and the production of succinic acid, formic acid and acetic acid. These experiments also showed that lactate, ethanol and fumaric acid production is too small to detect at the tested environmental conditions and could possibly be neglected. The values of these external fluxes are given in Table 2. To account for experimental error they are assumed have an accuracy of plus or minus 20%. A number of these values are given very small positive bounds (10^−4^-10^−8^) either because no flux was measured or because in the case of isocitrate we have assumed the flux towards it is positive with negligible magnitude (i.e. it is assumed to be consumed or removed by other processes not considered here).

**Table 2:** **Measured values of fluxes and assumed output of Isocitrate (see Figure** 2 **)**

**Reaction number**	**Flux values (mmol g-DCW** ^**−1**^ **h** ^**−1**^ **)**	**Comment**
1	7.457	Glycerol Input
14	0.000	Pyruvate Output
15	0.000	Fumarate Output
16	5.109	Succinate Output
17	0.000	Lactate Output
18	0.613	Formate Output
19	0.470	Acetate Output
20	0.000	Ethanol Output
34	0.000	Isocitrate Output
48	0.061	Biomass Production

To account for reversibility, the steps allowed to operate in both directions are split into forwards and backwards steps, ensuring that all reaction fluxes are constrained to have positive values. Also, a *practical* upper limit of 100 (mmol g-DCW^−1^ h^−1^) was set for all internal fluxes to prevent unfeasibly high fluxes occurring within cycles. In the cases where both forwards and backwards reaction steps have an upper limit of 100 it is possible that very high flux values could be obtained while the net flux is relatively low. Nevertheless, if further knowledge about these particular reactions is available, these “practical” upper limits can be appropriately reduced in order to more accurately constrain the computed solutions. For steps assumed to be irreversible a lower limit of 10^−8^ (mmol g-DCW^−1^ h^−1^) was implemented to remove the possibility of having an irreversible step with zero flux. This lower limit is implemented here because we envision using the flux distributions generated for our future work on this case in metabolic control analysis equations which include (1/flux) terms where very small values could cause numerical problems.

### Sampling results: the first flux distribution

**Figure 3:**
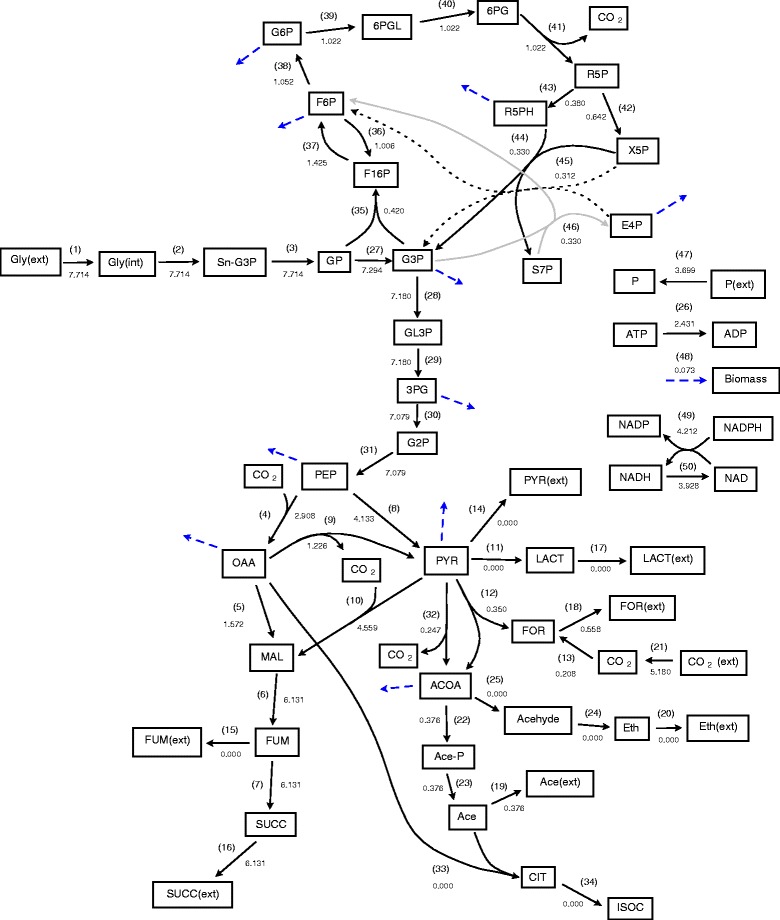
**The first flux distribution obtained through optimisation of equation** **4** **with F**_**pole**_ **= 0.** Values to 3 decimal places indicate corresponding flux values.

The first step in sampling is exactly equivalent to flux balance analysis, hence a linear objective for the system needs to be specified. Here we have set the linear objective to be the maximisation of succinic acid production (F_linear_ = v_16_, i.e. maximising reaction-16 flux (Succ→Succ(ext)) in Figure 2). Optimisation of equation 4 for this first flux distribution (where F_pole_=0) gives the flux distribution depicted in Figure 3. As it can be seen, the flux going to succinic acid (v_16_) has been maximised and a set of internal reaction fluxes which maintain this flux and the steady state of intermediates is shown. This includes a number of reaction fluxes with very low flux values, which in terms of the network could possibly be neglected. It is also interesting to note that this solution requires a large influx of CO_2_ and that a significant flux is needed going from pyruvate to malate to produce the maximal flux to succinic acid.

**Figure 4:**
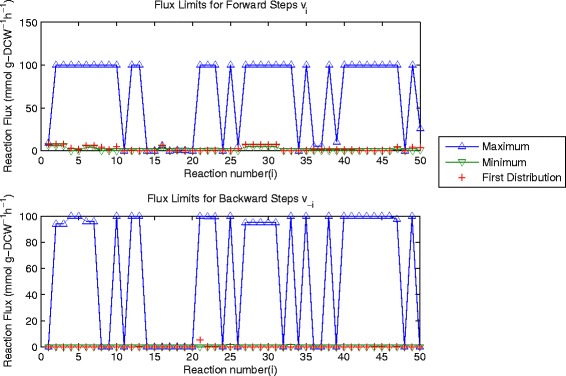
**Upper and lower limits for separate forwards and backwards reaction steps of the 1** ^**st**^ **flux distribution.** These values are computed with F_pole_ = 0.

Nevertheless, the solution depicted in Figure 3 is only one of many and the range of possible flux values can be demonstrated through flux variability analysis [9]. This can be accomplished through optimisation with multiple different linear objective functions. If reactions are split into forwards and backwards steps the complexity increases slightly, although the objective functions are still simple and straightforward. The upper and lower limits for reaction fluxes are determined using the objective functions *max*(*v*_i_) and *min*(*v*_i_) if the reactions are not separated. The upper and lower limits of the separate forwards and backwards reactions are determined with the optimisation functions *max*(*v*_i_), *min*(*v*_i_), *max*(*v*_−*i*_) and *min*(*v*_−*i*_). Here *v*_*i*_ is the flux through forwards reaction step *i* and *v*_−*i*_ is the flux through the backwards reaction –*i*. The overall flux limits are then defined through the objectives *max*(*v*_*i*_ - *v*_−*i*_) and *min*(*v*_*i*_ - *v*_−*i*_) It is worth noting that in this formulation *v*_*i*_ ≥ *0* and *v*_−*i*_ ≥ *0*. The ranges of possible fluxes are shown in Figure 4 which depicts the individual ranges of forwards and backwards steps and the overall limits for each reaction (forwards-backwards).

**Figure 5:**
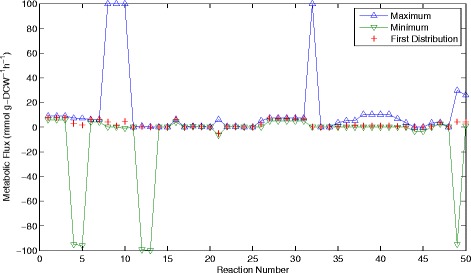
**Upper and lower limits for overall reaction fluxes (forwards-backward) of the 1** ^**st**^ **flux distribution.** These values are computed with F_pole_ = 0.

The range of possible solutions is defined by the areas between the upper and lower limits shown in Figures 4 and 5. Examining the values of the first flux distribution we can see that this single solution does not represent the full range of capabilities of this system. In Figure 4 we see that there is a very large range of possible solutions, partly due to the added search space introduced by considering separate forwards and backwards reactions for each step. Even if this is neglected, however, it is clear from Figure 5 that there are a number of reactions, which can have a large range of possible flux values. The largest of these are associated with branched and cyclical pathways, which allow many different solutions.

### Sampling results: second, third and fourth flux distributions

To generate alternative solutions we repeatedly optimise equations 2, 3, and 4 where F_pole_ ≠ 0 and where equations 2 and 3 include the values of previous solutions. For example the second flux distribution will be optimised using values from the first flux distribution (shown in Figure 3) in the poling penalty function (equation 2). As the number of existing solutions increases the complexity of these calculations will increase and the optimal value of F_pole_ is also expected to increase as it becomes more difficult for the optimiser to find new solutions that are sufficiently different from the previous ones.

**Figure 6:**
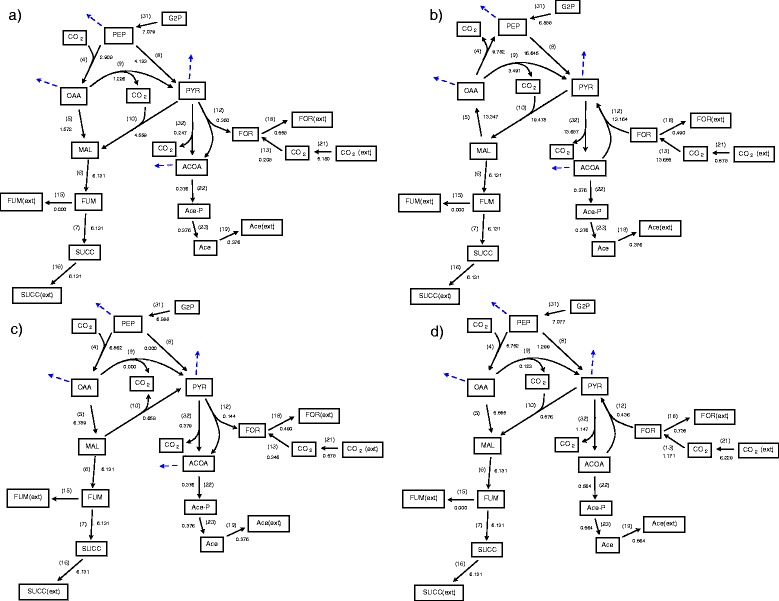
**Four flux distributions generated using optimisation of equations** **2** **,** **3** **and** **4** **with poling parameters N=2 and W**_**pole**_ **=1.** The first **(a)**, second **(b)**, third **(c)** and fourth **(d)** flux distributions are depicted on a significant subsection of the reaction network.

The values of fluxes obtained for the first four flux distributions generated using poling parameters N=2 and W_pole_=1 are shown in Figure 6. The second, third and fourth distributions obtained depend on the values of the poling parameters. They determine the relative weight of the poling penalty function in relation to the linear objective. In this case the linear objective is the maximisation of reaction-16 flux (flux 16, Succ→Succ(ext)) (the production of succinic acid) and it can be seen from Figure 6 that all the computed solutions obtain the same maximal value for flux 16. It is, however, clear that the internal reaction fluxes are different. In particular the fluxes through reactions connecting PEP, OAA, PYR and MAL and input of CO_2_ show significant differences. Figure 6 shows that fluxes 4, 10 and 12 (Malate dehydrogenase, ‘Malic’ enzyme and Pyruvate formate-lyase) can operate in different directions (see also Table 1 for allowed reversibilities) and reactions 8 and 9 (Pyruvate kinase and Oxaloacetate decarboxylase) can have zero flux while still achieving the same optimal succinic acid production. The value of CO_2_ uptake (flux 21, CO_2_(ext) ←→CO_2_) is also shown to vary significantly but in all four cases CO_2_ is always taken into the system through flux 21 and not released.

### Coverage analysis

The aim of sampling is to obtain a set of flux distributions, which represent all the possible combinations considering the limits shown in Figure 5. It is also desirable to maintain the optimal production of succinic acid but this is not guaranteed by the equations 2, 3, and 4 used in this optimisation. In other methods seeking alternative flux distributions a constraint is added to ensure the new solutions have either the same or similar values of the optimal fluxes [6,9]. Nevertheless, since both optimal and sub-optimal solutions (which can be obtained with and without the added constraint) are expected to provide interesting results we have aimed to reproduce these two types of solutions through optimisation of equations 2, 3, and 4 (with linear bias) to produce optimal values and through optimisation of equations 2, 3 and 5 (without linear bias) to produce both optimal and sub-optimal solutions. However, the inclusion of the linear objective (F_linear_ in equation 4) was found to generally give a hard constraint which excludes the possibility of finding near-optimal solutions (e.g. >95% of maximum yield) and instead only explores different optimal solutions (e.g. only computing combinations of flux distributions with the highest possible yield). To compute such near-optimal solutions we would recommend the addition of inequality constraints added to individual fluxes (e.g. >95% of maximum succinic acid production through reaction step 16) as opposed to modification of the objective function. The relative influence of the linear objective (F_linear_) and the poling-based penalty objective (F_pole_) depend on the values of the poling parameters (N and W_pole_). These values are chosen such that when the linear objective is included, all the solutions generated possess the optimal flux values (e.g. maximum yield). Nevertheless, the effect of these parameters on the optimisation performance as well as the resulting coverage of the sample generated are also explored. The coverage here shows how well the sample represents the range of possible functionality of the system. Samples are also compared against the linear objective (succinic acid production) to show if and how well the flux solutions meet this objective.

The coverage of the flux space (calculated with equations 6 and 7) increases with the number of flux distributions generated. If a high coverage is achieved then it can be assumed that the sample considers all possible options and hence this set can efficiently represent the system’s functionality. This methodology aims to achieve a high coverage with a minimum number of flux distributions. Alternative sampling methods [16-18] do not consider the coverage as an objective and may require very large numbers of iterations to achieve a high coverage. Also, combinatorial algorithms [10-14] do not consider the coverage and may generate impractically large numbers of solutions containing a high degree of redundancy.

**Figure 7:**
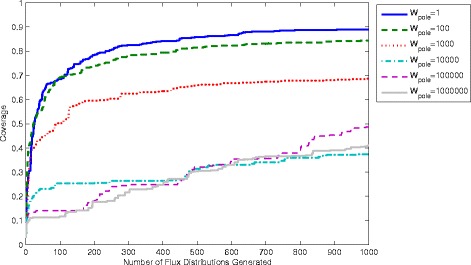
**Coverage computed for different values of the poling weight parameter W**_**pole**_ **with N=2.**

**Figure 8:**
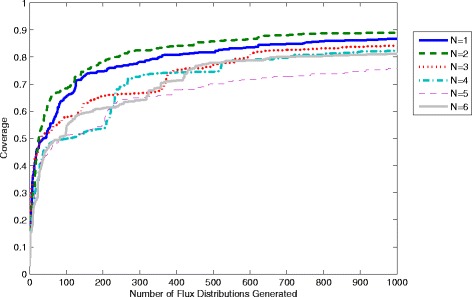
**Coverage computed for different values of the poling parameter N with W**_**pole**_ **=1.**

To demonstrate this methodology samples containing 1000 flux distributions have been generated using equations 2, 3 and 4 and different values of the poling parameters N and W_pole_. To compare the quality of samples coverage values are computed with 1–1000 distributions as shown in Figures 7 and 8. It is clear that the coverage increases as more flux distributions are generated. Using, however, different values for the parameters can give significantly different results. From Figure 7 we see that using a higher value of W_pole_ leads to samples with much lower coverage. This seems counter-intuitive since a higher value of W_pole_ gives more weight to the penalty function, which should lead to solutions with a greater difference. However, in practice this is shown to leave relatively large gaps in the coverage. This is possibly due to the “stiffness” introduced to the problem by a much larger magnitude penalty function, which leads to higher local optimum solutions. A global optimisation method would be able to find the ideal sample at the expense of greater computational time and effort. The optimisation method employed here is the SQP algorithm in the function fmincon within the software package “MatLab”. In Figure 8 the value of N is also shown to have an effect on the overall coverage obtained. Interestingly N=2 is shown to give the highest coverage, where N=1,3,4,5 and 6 all give lower coverage. Nevertheless, the difference is not as significant as that obtained using different values of W_pole._ As we can see all 6 samples give coverages between 0.75 and 0.9 (compared with 0.4 and 0.9 for different values of W_pole_).

**Figure 9:**
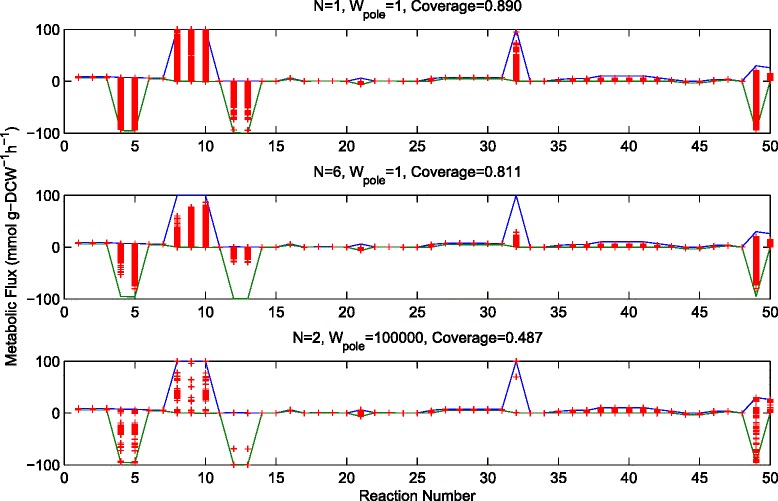
**Three samples generated using poling-based flux sampling with different values of N and W**_**pole**_ **.** For each sample 1000 flux distributions are generated.

The results of sampling are visualised in Figure 9, which depicts how three different samples are distributed within the possible flux space. Compared with the single distribution shown in Figure 5 it is clear that the distributions shown in Figure 9 better represent the range of capabilities of the system. In particular this is true for the samples having higher coverage, for example with lower values of W_pole_ and with N=2. It should be noted here that even the sample with the highest coverage still has gaps. This is expected since the optimisation function contains a linear objective (F_linear_) which is greater in magnitude than the poling penalty function (F_pole_) for all the samples generated. For example if the linear objective is the maximisation of a particular flux leading to a product, then the optimisation will avoid solutions which give sub-optimal production of that product (leaving a gap in the sub-optimal region). Hence, although the solutions are different they have an inherent linear bias pushing them in a particular direction.

### Analysis of the flux space: with and without linear bias

**Figure 10:**
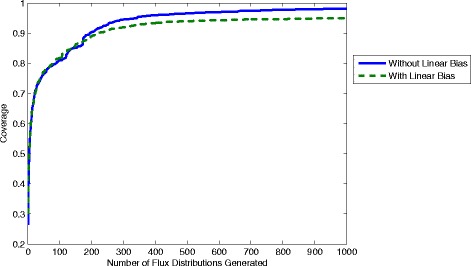
**Coverage computed with and without the linear objective using poling parameters W**_**pole**_ **=1 and N=2.**

To account for any influence from the linear element of the objective function we have also computed a sample using equations 2, 3 and 5. This involved using only the linear objective (F_total_ = F_linear_) to compute the first flux distribution then using only the poling penalty objective function (F_total_ = F_pole_) for the remaining 999 distributions. Hence the linear objective will not constrain or influence the resulting sample (which is equivalent to removing the linear bias). To compute “better” local optima, the optimisation is performed 10 times for each solution starting from random different initial conditions and keeping the solution with the smallest objective value (computed with equations 2, 3 and 5) to yield a higher overall coverage. A comparison of coverages computed with and without the linear objective is shown in Figure 10. In both cases the poling parameters are set to N=2 and W_pole_=1 and it is clear that the removal of the linear objective allows a higher coverage to be obtained.

**Figure 11:**
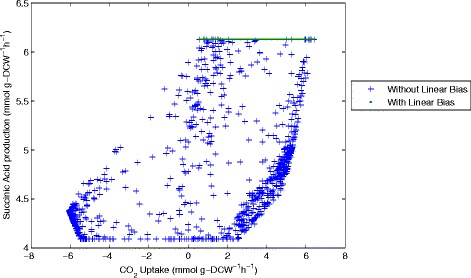
**CO**_**2**_ **uptake plotted against Succinic acid production (flux 16).** Samples computed with and without the linear objective are shown for comparison.

**Figure 12:**
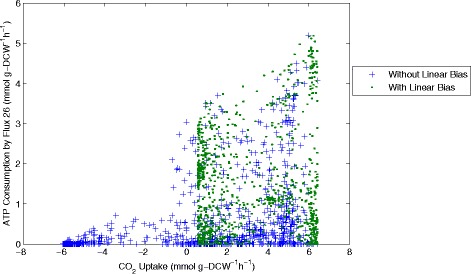
**CO**_**2**_ **uptake plotted against ATP consumption through flux 26.** Samples computed with and without the linear objective are shown for comparison.

**Figure 13:**
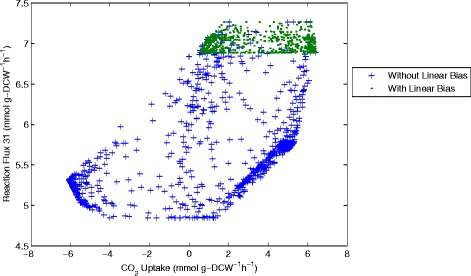
**CO**_**2**_ **uptake plotted against flux 31 (G2P → PEP).** Samples computed with and without the linear objective are shown for comparison.

**Figure 14:**
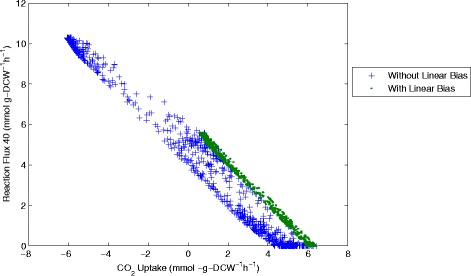
**CO**_**2**_ **uptake plotted against flux 40 (6PGL→6PG).** Samples computed with and without the linear objective are shown for comparison.

The results of sampling and the comparison with and without the linear objective can be seen more clearly when examining correlations between the different flux values. Representing results in this way shows a 2-dimensional slice of the possible solution space defined by the reaction network and the experimentally measured inputs and outputs. For example we can see the flux values of CO_2_ uptake (flux 21, CO_2_(ext)←→CO_2_) plotted against succinic acid production (flux 16, Succ→Succ(ext)) (Figure 11), ATP consumption through flux 26 (ATP→ADP + P) (Figure 12), an essential intermediate step in the glycolysis pathway (flux 31, G2P←→PEP) (Figure 13) and one step from the pentose-phosphate pathway (flux 40, 6PGL←→6PG) (Figure 14).

Analysis of the solution space identified with and without the linear objective in Figures 11, 12, 13 and 14 reveals several interesting features about the system. Importantly we can see that including the linear objective gives the maximum succinic acid production for all the solutions in the sample. If the linear objective is omitted a range of different possible rates of succinic acid production are obtained. Hence including the linear objective is equivalent to constraining the solution space to those providing the highest succinic acid production. An interesting point is that all the solutions, which yield the maximal succinic acid production also have a positive CO_2_ uptake through reaction 21 (CO_2_(ext)←→CO_2_). Solutions involving negative CO_2_ uptake (excretion) all have sub-optimal succinic acid production.

The ATP consumption through reaction 26 (ATP→ADP + P) refers to the energy requirements of the cell and it is intended to balance the ATP production in the rest of the reaction network. From the graph of CO_2_ uptake against ATP consumption through flux 26 we see that the solution space is constrained to a triangular region, which is further constrained in the sample generated with the linear objective. Both samples show that a higher CO_2_ uptake allows a higher consumption of ATP through reaction 26, although this is not a direct correlation as lower ATP consumption is also possible at higher CO_2_ uptake rates.

Another observed correlation is between reaction flux 40 (6PGL←→6PG) in the pentose-phosphate pathway and CO_2_ uptake. For the sample computed with the linear objective (which gives the highest succinic acid production) there appears to be an almost linear correlation between these two fluxes. This shows that to achieve the highest succinic acid production we either need a higher flux through the pentose phosphate pathway (which produces CO_2_) or a higher CO_2_ uptake rate. Without the linear objective, the shape of the solution space becomes more oval-shaped, but the same general correlation is maintained.

### Comparison with artificial centering hit and run algorithm

To compare our methodology with the existing methods based on Hit and Run algorithms we have sampled the above case study and two additional cases including a large synthetic reaction network and a genome-scale reaction network using the ACHR algorithm built into the toolbox COBRA [34,35].

It is worth noting that both sampling methods require similar pre-processes steps, both using a linear optimiser (for the smaller *Actinobacillus succinogenes* and synthetic cases linprog is used for both methods, and for the larger genome-scale model Gurobi [36] is used because it is able to handle the much larger number of linear constraints involved) to generate flux bounds and/or starting points for the sampler (called warm-up points in the case of the ACHR sampler). To make a fair comparison an equal number of warm-up points are used in both methods for each case considered, 200 points for the *Actinobacillus succinogenes* case, 4008 points in the case of the large synthetic reaction network and 4764 points in the case of the genome-scale reaction network.

Additional warm-up points could potentially increase the coverage achieved for both methods by providing improved starting points for the samplers, but at extra (at times significant) computational cost. We plan to explore this degree of freedom through full sensitivity analysis in a future publication, in connection with further development of the sampling methods.

For this comparison, the poling-based sampling is again using the SQP algorithm within the fmincon function inside MatLab. Here we have not used multiple restarts of the optimisation but instead we have increased the maximum number of function evaluations from 1000 to 10000 to give an improved convergence.

The ACHR sampler is run using different numbers of steps per set of fluxes stored which is a critical parameter affecting the quality of the sample generated and the CPU time required for sampling.

**Figure 15:**
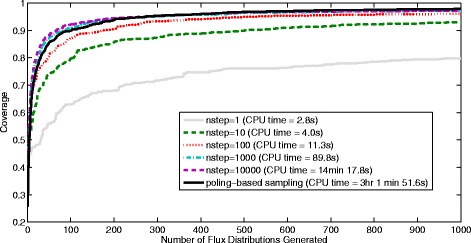
**Coverage obtained and CPU times required by the ACHR algorithm compared with poling-based sampling for the** ***Actinobacillus succinogenes*** **case.** The ACHR sampling is used here with different numbers of steps per record.

For the *Actinobacillus succinogenes* case 1000 flux distributions were contained in each sample. The coverage results computed with equations 6 and 7 can be seen in Figure 15 for each of the samples generated using both sampling methods (number of steps per record =1, 10, 100, 1000 and 10000 used for the ACHR sampler). Increasing the number of steps per record is shown to increase the coverage of the sample but also increases the computation time (having 10000 steps per record then requires 10^7^ flux distributions to be generated in total).

Comparing the coverage values obtained with the ACHR algorithm against those from those of our new method we can see in Figure 15 that if we have 1, 10 or 100 steps per record then the existing ACHR method does not perform as well as the new method proposed here (considering the optimal selection of N and W_pole_ without linear bias). Using 1000 or 10000 steps per record initially gives higher coverage than the poling-based method for the first ~200 flux distributions. However, the poling method is shown to achieve a slightly higher coverage than the ACHR algorithm as the number of distributions approaches 1000. For samples containing 1000 flux distributions the coverages obtained were 0.798, 0.930, 0.961, 0.973 and 0.973 for 1, 10, 100, 1000 and 10000 steps per record respectively. In comparison the sample generated with poling-based sampling gives a coverage of 0.979. If a global optimisation method had been used or if multiple restarts of the optimiation had be implemented then we expect that the coverage of the poling-based method could also have been higher than the step-wise approach taken by the ACHR method for the first 1~200 flux distributions. Although the ACHR algorithm does not guarantee a high coverage, it is intended to give a uniform coverage of the solution space and we can see that it will gradually achieve this as more flux distributions are added.

For these comparisons, all samples were generated using an Intel i5 3.40GHz desktop computer with 8Gb of memory. Considering the CPU time required for the sampling of the *Actinobacillus succinogenes* case it is clear that the ACHR method is much faster (approximately 13× faster) and can produce similar results if a high enough number of steps per record is chosen. This difference in computation times is mainly due to the time required by the non-linear optimisation required for the poling method which is much slower than a single step of the ACHR method. However, this is a small reaction network containing only 50 reaction steps and it is envisioned that the poling-based method will be more useful for larger genome-scale reaction networks containing 1000’s of reaction steps.

**Figure 16:**
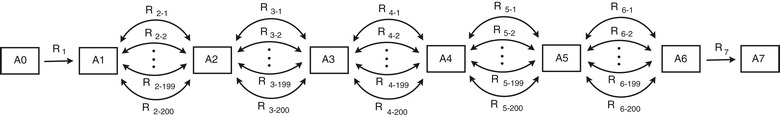
**Synthetic reaction network used for comparison of samplinjg methods at large scale.** This reaction network contains 7 metabolites labeled A0-A7 and 1002 reaction steps.

Hence, we have also considered the application of both sampling methods to a much larger synthetic reaction network containing 1002 reaction steps (shown in Figure 16) and to an even larger reaction network for the *E. coli* genome-scale reaction network iAF1260 [37]. To reduce the overall time required for the generation of multiple samples for these cases the number of flux distributions required has been reduced to 100.

**Figure 17:**
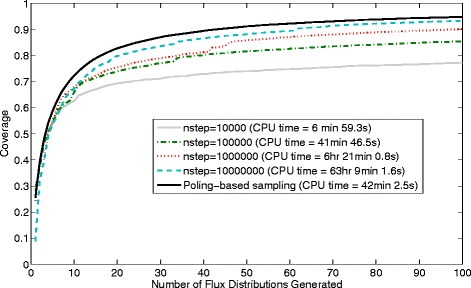
**Coverage obtained and CPU times required by the ACHR algorithm compared with poling-based sampling for the large synthetic reaction network case.** The ACHR sampling is used here with different numbers of steps per record.

In the synthetic reaction network the first reaction step (R_1_) is irreversible and constrained to a flux value of 1 mmol g-DCW^−1^ h^−1^ (plus or minus 20%) and the last step (R_7_) is also irreversible and constrained between 10^−8^ and 100 mmol g-DCW^−1^ h^−1^. The remaining 1000 reaction steps (including 5 sets of 200 parallel reactions) are reversible and they are split into forwards and backwards steps with flux ranges from 0 to 100 mmol g-DCW^−1^ h^−1^. Hence, this system can be used to test the two sampling methods. For this large synthetic case the ACHR method is implemented using 10000, 100000, 1000000 and 10000000 steps per record with the aim to achieve a high coverage in this large system. The poling-based method is run in exactly the same manner as for the *Actinobacillus succinogenes* case. A comparison of the resulting samples in Figure 17 shows that the poling-based method gives a higher coverage than the ACHR method, including samples generated with very large numbers of steps per record (up to 10000000 steps per record).

For this large reaction network case a comparison of the CPU times shows that the poling-based method is significantly faster (more than 60 × faster) than the ACHR method using the highest settings considered (10000000 steps per record) while still giving a higher coverage.

In the genome-scale model iAF1260 there are 2382 reaction steps, including 304 exchange reaction steps and 1 step for the creation of biomass. These exchange fluxes are specified to be constrained between +/− 0.1% of the values specified in the supplementary SBML file “Ec-1AF1260-flux1.xml” provided by Feist et al. [37]. In this case the reaction steps have not been split into forwards and backwards steps (to demonstrate that this is optional) and hence positive and negative values are allowed for reversible steps. Also, the maximum and minimum fluxes have been reduced from +99999/-99999 (specified in the SBML file of Feist et al. [37]) and +∞/-∞ (specified in the article of Feist et al. [37]) to +100/-100 mmol g-DCW^−1^ h^−1^ in order to avoid some of the very large flux values.

Due to the large number of linear constraints, linear optimisation (employing Gurobi) is used to provide an initial feasible point before the non-linear optimisation in the poling-based method. Otherwise the poling-based sampling is run in exactly the same way as with the two other cases.

For this genome-scale case the ACHR sampling method is implemented using 100000, 500000 and 1000000 steps per record with an aim to obtain a high coverage sample in this larger, more complex case.

**Figure 18:**
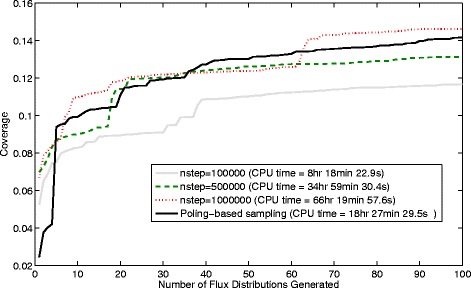
**Coverage obtained and CPU times required by the ACHR algorithm compared with poling-based sampling for the** ***E. coli*** **iAF1260 reaction network case.** The ACHR sampling is used here with different numbers of steps per record.

A comparison of the samples generated by both methods in Figure 18 shows that using 1000000 steps (at significant computational cost, see below) per record the ACHR method is able to generate samples with only slightly higher coverage than the poling method. However, the poling-based method produces much higher coverages than ACHR using 100000 steps per record and also higher than ACHR using 500000 steps per iteration.

It is worthwhile mentioning here that, as it can be seen in Figure 18, only low coverages can be obtained for this case with both our poling-based method and ACHR, which indicates that properly sampling the flux space of the iAF1260 network is a hard task.

Considering the CPU time for this genome-scale case it is clear that the poling-based method is beneficial as it requires only 18 hr 27 min 29.5 s which is much lower than the 66 hr 19 min 57.6 s and 34 hr 59 min 30.4 s times required for the two best solutions generated by the ACHR method. Additionally we believe that the CPU time for the poling-based method could be reduced much further by limiting the optimisation time to a maximum value and restarting, in the case of very slow line-search based convergence.

## Conclusions

A new methodology has been introduced to sample the possible flux space of biochemical systems. This is an extension of flux balance analysis, which involves the addition of a poling penalty function forcing new solutions away from any of the existing solutions generated. The resulting samples form a characteristic set of solutions, which can be used to analyse the space of possible solutions. An attractive feature of this approach is that solutions can be generated with and without the linear bias. Linear bias is the influence of the linear objective function on the sampling accomplished through multi-objective optimisation (linear objective + poling penalty function, equation 4). The addition of linear bias could also have been achieved through the addition of an extra constraint which is the method used for flux variability analysis [6] and [9]. However, in our approach the direct addition of linear bias to the objective function allows the sampling of solution space constrained to the optimal yield of succinic acid. Compared to sampling without linear bias we can see how the optimal flux space compares to the full space of possible fluxes including sub-optimal solutions.

We have also introduced new simple equations for measuring coverage, which illustrate how well a sample represents the range of possible solutions. Using these equations we have demonstrated that to obtain a sample without linear bias, increasing the weight of the poling function W_pole_ is not a good approach since this causes problems for the optimisation method which lead to inferior samples with poor coverage (see Figure 9). Instead, removing the linear objective after obtaining the 1^st^ solution in the sample (performing optimisation of equation 5) can give a higher coverage and a better sample covering the full space of possible solutions.

Plotting one reaction flux against another for the samples with and without linear bias is a very useful way to visualise the range of possible solutions. It can reveal if any correlations are present and importantly if there are some criteria or rules, which can be derived for obtaining the optimal flux values. This information is significant because it can be used to identify areas in the reaction network where the system is limited and how desired fluxes/yields can be enhanced. This also provides indications about how environmental conditions could be modified to enhance the production rates.

The addition of the poling penalty function makes the optimisation problem nonlinear. However, here we have performed the sampling employing deterministic, local optimisation methods which are more convenient for obtaining results in a shorter time. The use more intensive global optimisation methodologies (e.g. [38]) would lead to an improved coverage and a set of flux distributions which better represents the full range of possible solutions (possibly requiring higher computational effort).

Compared with the existing Artificial Centering Hit and Run algorithm [17] similar high coverages can be obtained for the *Actinobacillus Succinogenes* reaction network presented here. However, our method aims to obtain different solutions which maximise the coverage, while the ACHR algorithm is intended to give a uniform coverage but does not give high importance to the rate of convergence. For this reason the ACHR algorithm required the computation of 1000’s of steps per solution to obtain a high coverage for the *Actinobacillus succinogenes* reaction network case. ACHR sampling gives a relatively slow step-wise perturbation approach to covering the solution space, while our method approaches a higher coverage by obtaining very different solutions at every step. Hence we anticipate that our method will be more useful when applied to higher dimensional problems (e.g. genome-scale reaction networks) where the ACHR method may require a very large number of iterations to obtain a high coverage. This has been tested using a large synthetic reaction network where we show that the poling-based method can achieve a higher coverage than the ACHR algorithm, even when that algorithm is used with a very high number of steps per record (10000000). Additionally this has also been tested with a genome-scale *E. coli* reaction network iAF1260 [37] where it is found that the poling method can compute similar or better coverages thanACHR at a fraction of the computational cost. ACHR can obtain a slightly higher coverage only if a very high number of steps per record are used (1000000) at a significant additional computational cost.

A comparison of the CPU time required for the three cases (*Actinobacillus succinogenes,* the large synthetic reaction network and the *E. coli* genome-scale reaction network) shows that for cases where there is a small number of reaction steps (e.g. the *Actinobacillus succinogenes* case) the ACHR method is significantly faster while capable of achieving a similarly high coverage. However, if the number of reaction steps is very large (as in the genome-scale reaction network and the large synthetic reaction network cases) then (for the generation of a small representative set of flux distributions) the poling-based method can give a high coverage while requiring significantly less CPU time than a similar coverage sample generated using the ACHR method. In the case of the genome-scale reaction network the poling-based method is 2–3 times faster than the equivalent ACHR method. For the synthetic reaction network the CPU time of the poling-based method is more than 60 times faster than that of the equivalent ACHR method.

More generally the poling method [22] is good for sampling any problem involving very complex or large numbers of constraints provided that all the unknown parameters have finite bounds. It was originally developed for generating different 3D molecular conformations [22], which could be applied to problems such as 3D pharmacophore modelling [39] for reaction prediction and drug design. A feature of this type of sampling is that the number of calculations required for the penalty function will increase exponentially as the number of solutions grows larger. Hence it is inefficient to generate very large numbers of solutions this way. The problem with generating very large numbers of solutions is that when combined with sampling of other variables (e.g. elasticitiy coefficients [40-44]) the total number of combinations becomes impractically large. Hence it is preferable to capture all the characteristics of the system with the fewest flux distributions. In future versions of the sampling method we will attempt to modify this method so that solutions can be found quickly, even when the number of solutions grows large. Hence, generating of characteristic sets of solutions giving a high coverage should be possible in a very short time. The results of sampling the case study with *Actinobacillus succinogenes* are used in a companion paper (M. Binns, P. de Atauri, M. Cascante, C. Theodoropoulos) Sampling of systems with uncertain fluxes and elasticities using control bias as an indicator for targeted improvement of Actinobacillus succinogenes, in preparation) where flux sampling is exploited to investigate the metabolic control features of this organism at different flux states.

## Additional file

Additional file 1:
**The Additional file containing three tables with additional information about the reaction network of** ***Actinobacillus succinogenes*** **used in the case study.**

## Abbreviations

ACHR: Artificial centering hit-and-run (algorithm)
FBA: Flux balance analysis
MILP: Mixed integer linear programming
