# Supplementary material for: Sampling with poling-based flux balance analysis: optimal versus sub-optimal flux space analysis of Actinobacillus succinogenes
Source: BMC Bioinformatics. 2015 Feb 18;16:49. doi: 10.1186/s12859-015-0476-5 (PMC4350952; doi:10.1186/s12859-015-0476-5)
Supplement: Additional file 1: — The Additional file containing three tables with additional information about the reaction network of Actinobacillus succinogenes used in the case study. [file 12859_2015_476_MOESM1_ESM.pdf]

Appendix 1

The metabolic reaction network for *Actinobacillus succinogenes* consists of 50 reactions and 39 internal metabolites. The names and E.C. numbers of the enzymes involved are detailed below in table A1. Reaction flux bounds for this case study are given for forwards and backwards reaction steps in table A2 and the reaction stoichiometries are given in both tables A1 and A2. Additionally the common names for metabolites taken from the KEGG ligand database [30] are given below in table A3.

Table A1. Reactions numbers, enzyme E.C. numbers, reaction stoichiometries and enzyme common names for the *Actinobacillus succinogenes* reaction network.

Reaction number	Enzyme E.C. number	Reaction	Enzyme common name
1		Gly(ext) → Gly(int)	
2	2.7.1.30	Gly(int) + ATP ←→ ADP + sn-G3P	Glycerol Kinase
3	1.1.1.94	sn-G3P + NADP+ ←→ GP + NADPH	Glycerol-3-phosphate dehydrogenase
4	4.1.1.49	ADP + PEP + CO_2 ←→ ATP + OAA	Phosphoenolpyruvate carboxykinase (ATP)
5	1.1.1.37	NADH + OAA ←→ NAD+ + Mal	Malate dehydrogenase
6	4.2.1.2	Mal ←→ Fum	Fumarate hydratase
7	1.3.99.1	NADH + Fum ←→ NAD+ + Succ	Succinate dehydrogenase
8	2.7.1.40	ADP + PEP → ATP + Pyr	Pyruvate kinase
9	4.1.1.3	OAA → Pyr + CO_2	Oxaloacetate decarboxylase
10	1.1.1.40	NADPH + Pyr + CO_2 ←→ NADP+ + Mal	'Malic' enzyme
11	1.1.1.28	NADH + Pyr → NAD+ + Lact	D-lactate dehydrogenase
12	2.3.1.54	Pyr ←→ For + Acoa	Pyruvate formate-lyase
13	1.2.1.2	NAD+ + For ←→ NADH + CO_2	Formate dehydrogenase
14		Pyr → Pyr(ext)	
15		Fum → Fum(ext)	
16		Succ → Succ(ext)	
17		Lact → Lact(ext)	
18		For → For(ext)	
19		Ace → Ace(ext)	
20		Eth → Eth(ext)	
21		CO_2 ←→ CO_2(ext)	
22	2.3.1.8	Acoa + P ←→ Ace-P	Phosphate acetyltransferase
23	2.7.2.1	ADP + Ace-P ←→ ATP + Ace	Acetate kinase

24	1.1.1.1	NADH + Acehyde → NAD+ + Eth	Alcohol dehydrogenase
25	1.2.1.10	NAD+ + Acehyde ←→ NADH + Acoa	Acetaldehyde dehydrogenase
26		ATP → ADP + P	
27	5.3.1.1	GP ←→ G3P	Triose-phosphate isomerise
28	1.2.1.12	NAD+ + G3P + P ←→ NADH + GL3P	Glyceraldehyde-3-phosphate dehydrogenase
29	2.7.2.3	ADP + GL3P ←→ ATP + 3PG	Glycerate 3-phosphate kinase
30	5.4.2.1	3PG ←→ G2P	Phosphoglycerate mutase
31	4.2.1.11	G2P ←→ PEP	Phosphopyruvate hydratase
32	1.2.4.1, 2.3.1.12, 1.8.1.4	NAD+ + Pyr → NADH + CO_2 + Acoa	Pyruvate dehydrogenase complex
33	4.1.3.6	OAA + Ace ←→ Cit	Citrate lyase
34	4.2.1.3	Cit → Isoc	Aconitate hydratase
35	4.1.2.13	GP + G3P ←→ F16P	Fructose-bisphosphate aldolase
36	2.7.1.11	ATP + F6P → ADP + F16P	Fructose 6-phosphate kinase
37	3.1.3.11	F16P → P + F6P	Fructose 1,6-diphosphatase
38	5.3.1.9	F6P ←→ G6P	Glucose-6-phosphate isomerise
39	1.1.1.49	NADP+ + G6P → NADPH + 6PGL	Glucose-6-phosphate dehydrogenase
40	3.1.1.31	6PGL ←→ 6PG	6-phosphogluconolactonase
41	1.1.1.44	NADP+ + 6PG ←→ NADPH + CO2 + R5P	Phosphogluconate dehydrogenase
42	5.1.3.1	R5P ←→ X5P	D-ribulose 5-phosphate epimerase
43	5.3.1.6	R5P ←→ R5PH	Ribose-5-phosphate isomerise
44	2.2.1.1	G3P + S7P ←→ X5P + R5PH	Glycolaldehydetransferase
45	2.2.1.1	G3P + F6P ←→ X5P + E4P	Glycolaldehydetransferase
46	2.2.1.2	G3P + S7P ←→ F6P + E4P	Transaldolase
47		P ←→ P(ext)	
48		46.93 ATP + 13.439 NADPH + 2.727 NAD+ + 0.099 G3P + 0.528 PEP + 1.502 OAA + 2.764 Pyr + 3.006 Acoa + 1.37 3PG + 0.126 F6P + 0.41 G6P + 0.686 R5PH + 0.244 E4P → 46.93 ADP + 13.439 NADP+ + 2.727 NADH + biomass	
49	1.6.1.2	NADPH + NAD+ ←→ NADP+ + NADH	NAD(P)+ transhydrogenase
50		NADH → NAD	

Table A2. Reactions numbers, reaction stoichiometries and flux bounds for forwards and backwards reaction steps in the *Actinobacillus succinogenes* reaction network.

Reaction number	Reaction (→ = forwards, ← = backwards)	Forwards reaction flux bounds		Backwards reaction flux bounds	
		Lower bound	Upper bound	Lower bound	Upper bound
1	Gly(ext) → Gly(int)	5.9659	8.9488	0	0
2	Gly(int) + ATP ←→ ADP + sn-G3P	0	100	0	100
3	sn-G3P + NADP+ ←→ GP + NADPH	0	100	0	100
4	ADP + PEP + CO_2 ←→ ATP + OAA	0	100	0	100
5	NADH + OAA ←→ NAD+ + Mal	0	100	0	100
6	Mal ←→ Fum	0	100	0	100
7	NADH + Fum ←→ NAD+ + Succ	0	100	0	100
8	ADP + PEP → ATP + Pyr	1.0000e-08	100	0	0
9	OAA → Pyr + CO_2	1.0000e-08	100	0	0
10	NADPH + Pyr + CO_2 ←→ NADP+ + Mal	0	100	0	100
11	NADH + Pyr → NAD+ + Lact	1.0000e-08	100	0	0
12	Pyr ←→ For + Acoa	0	100	0	100
13	NAD+ + For ←→ NADH + CO_2	0	100	0	100
14	Pyr → Pyr(ext)	1.0000e-08	0.0001	0	0
15	Fum → Fum(ext)	1.0000e-08	0.0001	0	0
16	Succ → Succ(ext)	4.0872	6.1307	0	0
17	Lact → Lact(ext)	1.0000e-08	0.0001	0	0
18	For → For(ext)	0.4902	0.7353	0	0
19	Ace → Ace(ext)	0.3757	0.5636	0	0
20	Eth → Eth(ext)	1.0000e-08	0.0001	0	0
21	CO_2 ←→ CO_2(ext)	0	100	0	100
22	Acoa + P ←→ Ace-P	0	100	0	100
23	ADP + Ace-P ←→ ATP + Ace	0	100	0	100
24	NADH + Acehyde → NAD+ + Eth	1.0000e-08	100	0	0
25	NAD+ + Acehyde ←→ NADH + Acoa	0	100	0	100
26	ATP → ADP + P	1.0000e-08	100	0	0
27	GP ←→ G3P	0	100	0	100
28	NAD+ + G3P + P ←→ NADH + GL3P	0	100	0	100
29	ADP + GL3P ←→ ATP + 3PG	0	100	0	100
30	3PG ←→ G2P	0	100	0	100
31	G2P ←→ PEP	0	100	0	100
32	NAD+ + Pyr → NADH + CO_2 + Acoa	1.0000e-08	100	0	0
33	OAA + Ace ←→ Cit	0	100	0	100
34	Cit → Isoc	1.0000e-08	0.0001	0	0
35	GP + G3P ←→ F16P	0	100	0	100
36	ATP + F6P → ADP + F16P	1.0000e-08	100	0	0
37	F16P → P + F6P	1.0000e-08	100	0	0
38	F6P ←→ G6P	0	100	0	100
39	NADP+ + G6P → NADPH + 6PGL	1.0000e-08	100	0	0
40	6PGL ←→ 6PG	0	100	0	100
41	NADP+ + 6PG ←→ NADPH + CO2 + R5P	0	100	0	100
42	R5P ←→ X5P	0	100	0	100
43	R5P ←→ R5PH	0	100	0	100
44	G3P + S7P ←→ X5P + R5PH	0	100	0	100
45	G3P + F6P ←→ X5P + E4P	0	100	0	100
46	G3P + S7P ←→ F6P + E4P	0	100	0	100
47	P ←→ P(ext)	0	100	0	100
48	46.93 ATP + 13.439 NADPH + 2.727 NAD+ + 0.099 G3P + 0.528 PEP + 1.502 OAA + 2.764 Pyr + 3.006 Acoa + 1.37 3PG + 0.126 F6P + 0.41 G6P + 0.686 R5PH + 0.244 E4P → 46.93 ADP + 13.439	0.0489	0.0734	0	0

	NADP+ + 2.727 NADH + biomass				
49	NADPH + NAD+ ←→ NADP+ + NADH	0	100	0	100
50	NADH → NAD	1.0000e-08	100	0	0

Table A3. Common names for the metabolites involved in the *Actinobacillus succinogenes* reaction network.

Metabolite	Common name
Gly(int)	Glycerol (internal)
Sn-G3P	sn-Glycerol 3-phosphate
GP	Glycerone Phosphate
G3P	D-Glyceraldehyde 3-phosphate
PEP	Phosphoenolpyruvate
OAA	Oxaloacetate
MAL	(S)-Malate
FUM	Fumarate
SUCC	Succinate/Succinic acid
PYR	Pyruvate
LACT	D-Lactate
FOR	Formate/Formic acid
CO_2	Carbon dioxide
ACOA	Acetyl-CoA
Ace	Acetate
Eth	Ethanol
Ace-P	Acetyl phosphate
Acehyde	Acetaldehyde
GL3P	3-Phospho-D-glyceroyl phosphate
P	Orthophosphate
3PG	3-Phospho-D-glycerate
G2P	2-Phospho-D-glycerate
CIT	Citrate
F16P	Beta-D-Fructose 1,6-bisphosphate
F6P	D-Fructose 6-phosphate
G6P	D-Glucose 6-phosphate
6PGL	D-Glucono-1,5-lactone 6-phosphate
6PG	6-Phospho-D-gluconate
R5P	D-Ribulose 5-phosphate
X5P	D-Xylulose 5-phosphate
R5PH	D-Ribose 5-phosphate
S7P	Sedoheptulose 7-phosphate
E4P	D-Erythrose 4-phosphate
ATP	Adenosine 5'-triphosphate
ADP	Adenosine 5'-diphosphate
NAD+	Nicotinamide adenine dinucleotide
NADH	Reduced nicotinamide adenine dinucleotide
NADP+	Nicotinamide adenine dinucleotide phosphate
NADPH	Reduced nicotinamide adenine dinucleotide phosphate
